# The Spanish Uniform Newborn Screening Panel (SUSP): A National Consensus Framework for Harmonized Newborn Screening

**DOI:** 10.3390/ijns12030067

**Published:** 2026-08-13

**Authors:** Judit García-Villoria, Rosa María López-Galera, Carmen Delgado-Pecellín, Dolores Rausell Félix, Cristóbal Colón, Hugo Rocha, Belén Pérez, Antonia Ribes, María Luz Couce, Domingo González-Lamuño

**Affiliations:** 1 Division of Inborn Errors of Metabolism-IBC, Department of Biochemistry and Molecular Genetics, Biomedical Diagnostic Center, Hospital Clinic of Barcelona, 08028 Barcelona, Spain; rmlopez@clinic.cat (R.M.L.-G.); aribes@clinic.cat (A.R.); 2 Fundació de Recerca Clínic Barcelona-Institut d’Investigacions Biomèdiques August Pi i Sunyer (FCRB-IDIBAPS), 08036 Barcelona, Spain; 3 Centro de Investigación Biomédica en Red de Enfermedades Raras (CIBERER), ISCIII, 28029 Madrid, Spain; bperez@cbm.csic.es (B.P.); maria.luz.couce.pico@sergas.es (M.L.C.); 4 Division of Metabolic Disorders Unit, Department of Biochemistry, Hospital Universitario Virgen del Rocio, IBIS, 41013 Sevilla, Spain; carmen.delgado.sspa@juntadeandalucia.es; 5 Laboratory of Metabolic Disorders, Department of Clinical Analysis, Hospital Universitari i Politècnic La Fe, 46026 Valencia, Spain; rausell_dol@gva.es; 6 Laboratory of Metabolic Disorders, Unit for the Diagnosis and Treatment of Congenital Metabolic Diseases, Spanish (CSUR) and European (MetabERN) Reference Center, Clinical University Hospital of Santiago de Compostela, 15706 Santiago de Compostela, Spain; cristobal.colon.mejeras@sergas.es; 7 Newborn Screening, Metabolism and Genetics Unit, Human Genetics Department of the National Institute of Heath Doutor Ricardo Jorge, 4000-055 Porto, Portugal; hugo.rocha@insa.min-saude.pt; 8 Centro de Diagnóstico de Enfermedades Moleculares, Centro de Biología Molecular, Raras, IdiPAZ, Universidad Autónoma de Madrid, 28049 Madrid, Spain; 9 Neonatology Department, Metabolic Unit, RICORS-SAMID, University Clinical Hospital of Santiago de Compostela, Health Research Institute of Santiago de Compostela (IDIS), 15706 Santiago de Compostela, Spain; 10 Division of Pediatrics, University Hospital Marqués de Valdecilla, University of Cantabria and Research Insitiute Valdecilla (IDIVAL), 39008 Santander, Spain

**Keywords:** genomic newborn screening, health equity, inborn errors of metabolism, national consensus, newborn screening, primary and secondary conditions, public health policy, Spain

## Abstract

Newborn screening (NBS) is a cornerstone of preventive medicine, enabling early diagnosis and treatment of severe congenital disorders. In Spain, despite the existence of a Basic Common Portfolio, the absence of a harmonized national panel has led to significant inter-regional variability, affecting equity in access to early diagnosis. The Spanish Uniform Screening Panel (SUSP) was developed through a structured nationwide consensus process involving all NBS centers in Spain, with additional input from Portugal. The process included a comprehensive survey of current practices, expert workshops, multiple consensus rounds, and predefined inclusion criteria. Disease nomenclature was standardized using OMIM and ORPHAcode identifiers. Conditions were classified as primary screening targets or secondary findings, and consensus was reached on confirmatory biochemical and genetic testing pathways. The operational dataset comprised 48 biomarker-based screening entries. Because some conditions can be detected through multiple primary markers, duplicate analytical routes were retained but counted once, resulting in a total 189 unique clinical conditions, including 87 primary and 102 secondary conditions. The SUSP represents the first nationwide harmonized framework for newborn screening in Spain and constitutes a major step toward equitable and standardized implementation. It provides a scalable model that may inform other countries facing similar disparities.

## 1. Introduction

Newborn screening (NBS) programs are designed to identify severe congenital conditions early in life, enabling timely interventions that prevent irreversible morbidity and mortality. Spain has notable strengths in diagnosing and managing inborn errors of metabolism, building on pioneering efforts initiated in the 1960s and further advanced by the adoption of tandem mass spectrometry in the 2000s [1]. A national network of specialized centers contributes to the delivery of high-quality care and ongoing monitoring of rare diseases [2].

Despite these strengths, Spain has a national Basic Common Portfolio for NBS, but lacks a unified comprehensive expanded panel implemented uniformly across all autonomous communities (ACs). In contrast to the U.S. Recommended Uniform Screening Panel (RUSP) [3]—which has recently faced significant central governance restructuring—and the Italian Extended Neonatal Screening (ENS) program [4,5], the Spanish system remains fragmented, with marked variability across ACs in the number of conditions screened and the terminology used.

**Figure 1:**
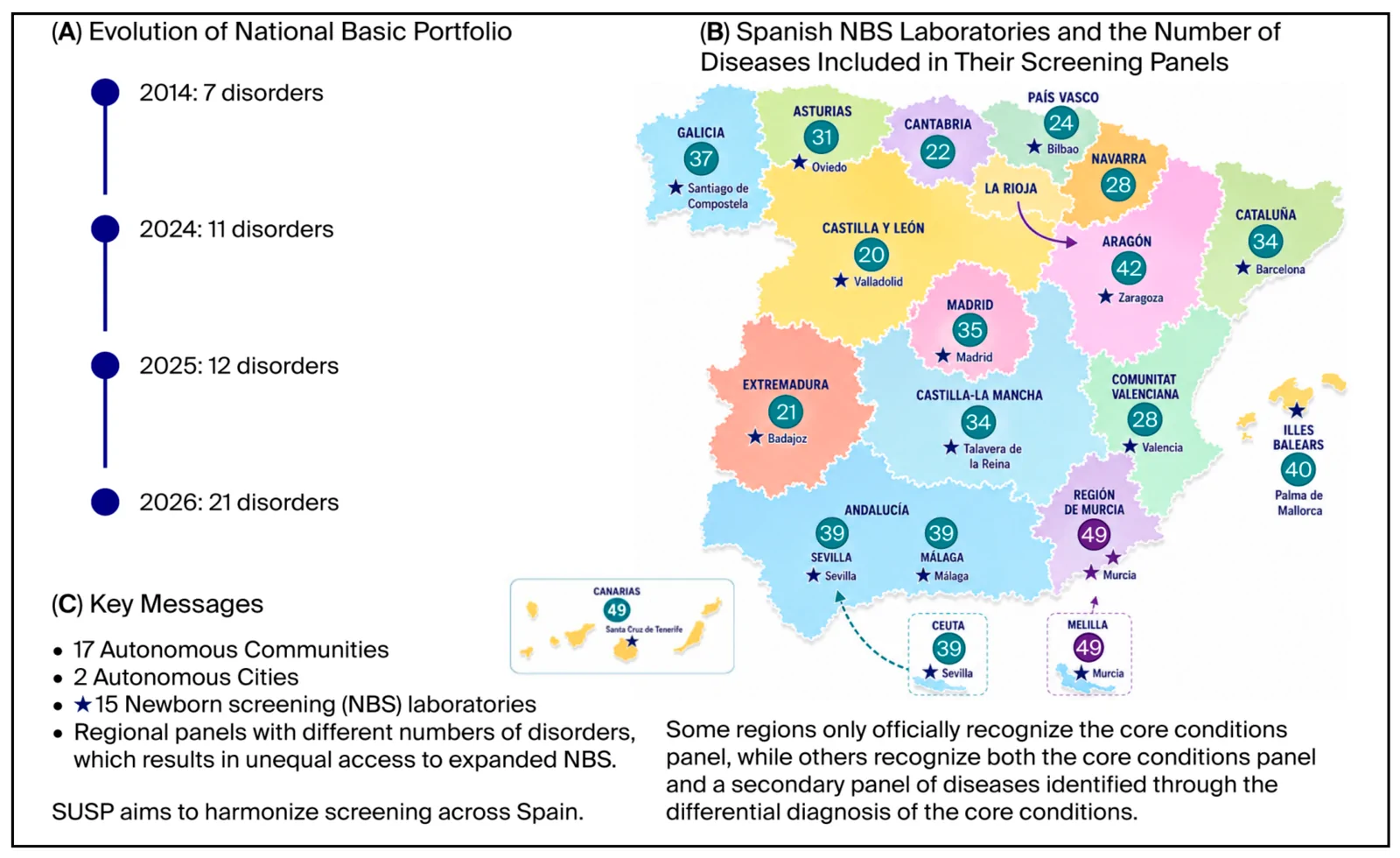
Newborn screening programs across the Spanish Autonomous Communities in 2026, before implementation of the Spanish Uniform Screening Panel (SUSP). (**A**) Evolution of the Spanish National Basic Common Portfolio from 2014 to 2026. (**B**) Number of disorders included in each regional newborn screening program in 2026. (**C**) Summary of the Spanish newborn screening network. The figure illustrates the marked regional heterogeneity that existed before development of the SUSP and the resulting inequalities in access to expanded newborn screening.

Since 2014, national regulation has established a Basic Common Portfolio for NBS. In 2025, the incorporation of additional conditions was approved, aiming to expand the common program to 21 conditions, although implementation and regulatory rollout require clarification according to the applicable national framework. However, each autonomous community retains the authority to extend its panel, resulting in substantial heterogeneity. Therefore, depending on the AC, screening panels currently range from the national minimum of 21 conditions to more than 40, reflecting differences in regional implementation policies rather than scientific criteria alone. Consequently, access to early diagnosis and treatment may vary according to the newborn’s place of birth. Figure 1 illustrates the current geographical distribution of screening panels across Spain. This variability affects not only the number of disorders included but also disease nomenclature, diagnostic pathways, and reporting standards, ultimately compromising equity in neonatal healthcare [6].

In response to this scenario, a multidisciplinary group developed a harmonized national consensus panel with standardized nomenclature and diagnostic pathways: the Spanish Uniform Screening Panel (SUSP).

## 2. Materials and Methods

The SUSP was specifically developed to harmonize disorders detected through laboratory-based dried blood spot (DBS) newborn screening. This focus was deliberately chosen because DBS-based screening is currently the area showing the greatest heterogeneity among the Spanish ACs. In contrast, other newborn screening programmes, such as universal newborn hearing screening and pulse oximetry screening for critical congenital heart disease, are already implemented nationwide according to standardized protocols and were therefore considered outside the scope of this consensus.

### 2.1. Expert Panel and Consensus Process

The SUSP was developed by a multidisciplinary expert panel comprising 45 professionals; most of them were members of the Neonatal Screening Working Group of the Spanish Society for the Study of Inborn Errors of Metabolism (AECOM). The panel included clinicians, biochemists, geneticists, and researchers involved in newborn screening programs. In addition, it included members of the Spanish Network for Biomedical Research on Rare Diseases (CIBERER). Importantly, each Spanish newborn screening laboratory was represented by at least one laboratory director or senior specialist, ensuring nationwide representation of all regional newborn screening programs.

Portugal was included because of its longstanding scientific collaboration with the AECOM Working Group, providing valuable expertise from a comparable healthcare context within the Iberian Peninsula.

### 2.2. Consensus Process

The consensus methodology consisted of five sequential phases: (i) A nationwide survey of all newborn screening laboratories to document current screening panels, biomarkers, and laboratory practices; (ii) A comprehensive review of the scientific literature and international newborn screening recommendations; (iii) Three structured voting rounds were performed. Consensus was defined a priori as ≥80% agreement. Items not reaching consensus were discussed during online meetings and resubmitted until agreement was achieved; (iv) Consensus on disease classification, standardized nomenclature (OMIM and ORPHANET), biomarkers, biochemical and genetic confirmatory testing; and (v) Final review and approval of the Spanish Uniform Screening Panel (SUSP) by all participating experts.

Conditions were eligible for inclusion only if validated biomarkers were already implemented in Spanish NBS laboratories. Inclusion criteria are summarized in Table 1. These criteria were selected as the minimum operational requirements applicable to laboratory-based newborn screening and do not intend to reproduce the complete Wilson and Jungner framework.

Each disorder was individually evaluated by the expert panel against the predefined inclusion criteria and subsequently classified by consensus as either a primary or secondary condition. Disease nomenclature was harmonized using OMIM and ORPHA code identifiers. The distinction between primary and secondary conditions was based on predefined clinical and technical criteria rather than solely on biomarker detectability. Accordingly, conditions were classified into two categories:

Primary or Core Conditions: Disorders that fulfill the criteria defined in Table 1 and were designated as the direct targets of newborn screening.

Secondary Conditions: Disorders not intentionally targeted as primary screening conditions but that may be detected through the same biomarker or analytical pathway used for a core condition. These diseases are identified incidentally during the differential diagnosis of a core condition, but do not fulfill the predefined criteria to be included as primary condition, either because no effective treatment is available, the disorder is not considered sufficiently severe, or the biomarker lacks adequate sensitivity or specificity.

Consensus was also reached regarding the recommended biochemical and molecular confirmatory diagnostic pathways for each primary screening biomarker.

Conditions identifiable through more than one primary biomarker were retained as separate operational entries in the dataset to reflect their distinct biomarker-based screening pathways, but each condition was counted only once in the final disease totals.

## 3. Results

Based on the predefined criteria described in the Section 2, the national consensus process resulted in the development of the Spanish Uniform Screening Panel (SUSP), comprising 189 unique clinical conditions: 87 classified as primary conditions and 102 as secondary conditions. Primary conditions are disorders that fulfil the predefined inclusion criteria and constitute the intended targets of newborn screening, whereas secondary conditions are disorders identified during the differential diagnostic evaluation triggered by an abnormal first-tier screening biomarker. All conditions were assigned standardized nomenclature using OMIM and ORPHANET identifiers, and expert consensus defined the corresponding biochemical and genetic confirmatory diagnostic pathways associated with each first-tier screening marker. The complete panel is presented in Table 2, Table 3, Table 4, Table 5, Table 6, Table 7 and Table 8. The panel reflects current newborn screening practice in Spain and includes only conditions for which validated screening biomarkers are already implemented in routine newborn screening.

The tables should be interpreted as a standardized framework for the diagnostic evaluation initiated after an abnormal newborn screening result rather than as complete newborn screening algorithms. Disorders are grouped according to the first-tier screening biomarker identified in the dried blood spot (DBS) specimen. All biomarkers routinely monitored in newborn screening laboratories are included in the SUSP, even when their clinical interpretation depends on their combination with other biomarkers rather than on an isolated abnormality. Consequently, some biomarkers may be associated only with secondary conditions when considered in isolation, while constituting part of a primary screening pattern when interpreted together with other routinely monitored biomarkers.

To maintain consistency with the current organizational framework of the Spanish Ministry of Health, disorders are first presented according to the official “Condition or Group of Conditions” classification used in the national newborn screening programme. The adjacent “Condition” column specifies the individual disorders included within each group, thereby providing the level of detail required for standardized biochemical and molecular diagnostic evaluation.

The remaining columns summarize the consensus recommendations for the post-screening diagnostic work-up, including the recommended biochemical or functional confirmatory investigations and the diagnostic gene panel to be considered for molecular differential diagnosis. The “Associated Disease Gene(s)” column identifies the causative gene(s) for each individual disorder, whereas the “Recommended Diagnostic Gene Panel” represents the complete set of genes that should be considered during the molecular diagnostic evaluation initiated by a specific abnormal screening biomarker. Because several disorders may share overlapping biochemical profiles or screening biomarkers, this panel may include genes associated with conditions listed elsewhere in the tables. Consequently, the recommended diagnostic gene panel should not be interpreted as a second-tier screening strategy but as part of the confirmatory diagnostic evaluation following an abnormal newborn screening result.

For conditions characterized by marked genetic heterogeneity, particularly primary immunodeficiencies, the genes listed correspond to those recommended for molecular diagnostic confirmation following an abnormal screening result and subsequent clinical evaluation. In these disorders, the primary screening biomarker identifies newborns requiring further diagnostic evaluation rather than a single disease entity. Consequently, several clinically distinct disorders may be diagnosed through the same confirmatory genetic analysis. Within the SUSP, only disorders fulfilling the predefined inclusion criteria are classified as primary conditions, whereas additional disorders identified during this standardized diagnostic evaluation are classified as secondary conditions.

Finally, the consensus proposal has been presented to the Spanish Ministry of Health with the objective of providing a scientifically robust framework for the harmonization of NBS programs across all the ACs [8].

**Table 8:** Other disorders: Primary and secondary conditions (PC and SC) in the Spanish Uniform Screening Panel (SUSP), grouped by primary marker detected by newborn screening.

First-tier Screening Biomarker (DBS)	Official Condition or Group of Conditions (Spanish Ministry of Health)	Condition	ORPHA Code	Consensus 1: PC 2: SC	OMIM Disease Number	Associated Disease Gene(s) *	OMIM Gene Number	Recommended Biochemical or Functional Confirmatory Tests	Recommended Gene Panel for Differential Molecular Diagnosis **	RUSP 1: PC 2: SC	Portugal 1: PC 2: SC
Deletion exon 7 of *SMN1*	Spinal muscular atrophy (SMA) #	Spinal muscular atrophy (SMA)	70	1	253300/ 253550/ 253400/ 271150	*SMN1*	600354	*SMN1* gene analysis and *SMN2* copy number determination	*SMN1*	1	1
IRT ↑	Cystic fibrosis (CF) #	Cystic fibrosis (CF)	586	1	219700	*CFTR*	602421	Sweat test	*CFTR*	1	1
TREC, KREC ↓	Severe combined immunodeficiency (SCID) #	Severe combined immunodeficiency (SCID)	183660	1	1027007 other	More than 300 genes [4]	NA	T-cell functional assays and T, B, and NK cell counts by flow cytometry	More than 300 genes [4]	1	N.I.
	Other immunodeficiencies	X-linked agammaglobulinemia (XLA), KREC ↓	47	2	300755/ 30310	*BTK/* *SH3KBP1*	300300/ 300374	T-cell functional assays and T, B, and NK cell counts by flow cytometry	*BTK/* *SH3KBP1*	1	N.I.
		DiGeorge syndrome	567	2	188400	*TBX1*	602054	T-cell functional assays and T, B, and NK cell counts by flow cytometry	*TBX1*	1	N.I.
		Ataxia–telangiectasia	100	2	208900	*ATM*	607585	T-cell functional assays and T, B, and NK cell counts by flow cytometry	*ATM*	1	N.I.
		Other non-severe combined immunodeficiencies	None	2	NA	More than 300 genes [9]	NA	T-cell functional assays and T, B, and NK cell counts by flow cytometry	More than 300 genes [9]	1	N.I.

↑ Increased; ↓: Decreased; #: Disease included in Basic Common Portfolio in Spain.; * “Associated Disease Gene(s)” identifies the causative gene(s) for each individual disorder; ** “Recommended Gene Panel for Differential Molecular Diagnosis” lists the genes that should be considered during the molecular diagnostic evaluation initiated by an abnormal primary screening biomarker, thereby facilitating the differential diagnosis of disorders with overlapping biochemical profiles; DBS: dried blood spot; IRT: Immunoreactive trypsinogen; N.I.: not included; PC: primary condition; RUSP: Recommended Uniform Screening Panel from the U.S.; SC: Secondary condition; TREC and KREC: T-cell and B-cell receptor excision circles; After a literature review focused on international newborn screening recommendations, previously published consensus documents, evidence regarding the clinical benefit of early diagnosis, and analytical performance of screening biomarkers, mucopolysaccharidosis type II (MPS-II), acid sphingomyelinase deficiency (ASMD), metachromatic leukodystrophy (MLD), alpha-mannosidosis, Krabbe disease, aromatic L-amino acid decarboxylase (AADC) deficiency, phosphoglycerate dehydrogenase deficiency, succinic semialdehyde dehydrogenase deficiency, and cerebral creatine deficiency syndrome were excluded, but designated for future evaluation once sufficient evidence becomes available. Their future inclusion will require feasibility assessments, biomarker validation in national reference laboratories, and formal review by the national advisory board before incorporation into the SUSP.

## 4. Discussion

The SUSP represents the first national framework for consensus on newborn screening in Spain, addressing long-standing regional disparities in access to early diagnosis and treatment. If adopted nationwide, it has the potential to ensure that Spanish newborns, regardless of their region of birth, receive equitable access to preventive care.

A key methodological strength of this work is the distinction between biomarker-based screening entries and unique clinical conditions. This approach reflects real-world laboratory practice, where multiple biomarkers may detect the same disorder, and enhances methodological transparency and reproducibility across screening programmes.

The geographical variability shown in Figure 1 clearly illustrates that access to expanded newborn screening remains dependent on the AC where a child is born. Reducing these territorial inequalities represents one of the main objectives of the SUSP and reinforces its importance as a public health initiative aimed at ensuring equitable access to early diagnosis, confirmatory testing, and appropriate clinical management throughout Spain.

The SUSP can be contextualized within international newborn screening frameworks such as the U.S. RUSP [3], the Italian ENS program [4,5], and the Portuguese national screening program. While the RUSP provides a centrally defined, evidence-based framework with a set of core conditions, its implementation occurs within a federal system where states retain flexibility. Furthermore, the RUSP model has recently demonstrated structural vulnerability following the Department of Health and Human Services decision to dissolve its longstanding Scientific Advisory Committee, shifting newborn screening expansions toward direct executive interventions and leaving a procedural void in standardized evidence review. In contrast, the Portuguese program represents a highly centralized model with uniform nationwide implementation. The SUSP occupies an intermediate position, combining a decentralized healthcare structure with a consensus-driven effort toward national harmonization. This hybrid approach may offer a particularly relevant pathway for countries with regional variability, balancing scientific standardization and institutional stability with local implementation capacity.

Although several European countries have expanded their newborn screening programmes during the last decade [4], considerable variability remains regarding the number of screened conditions, disease nomenclature, laboratory methodologies, and governance models. Recent European initiatives have highlighted the importance of harmonization, standardized nomenclature, common quality standards, and collaborative data sharing to improve equity and facilitate comparison of programme outcomes across countries. In this context, the SUSP represents a structured national framework that is fully aligned with these broader European objectives while addressing the specific needs of the Spanish healthcare system.

As in Italy, where national legislation is supported by scientific review and regional readiness, the Spanish model emphasizes quality and feasibility over quantity. This approach is particularly relevant in the context of emerging multiplex technologies, such as enzymatic assays for lysosomal storage disorders, which are likely to identify additional secondary conditions and therefore require dynamic updates of the panel.

This consensus also aligns with international recommendations that call for greater harmonization of newborn screening policies. The International Society for Neonatal Screening (ISNS) and the World Health Organization (WHO) have emphasized the importance of equity, standardized disease nomenclature, and evidence-based expansion of screening programs. Furthermore, European Regulation (EU) 2021/2282 on Health Technology Assessment (HTA) provides a broader framework for transparency and joint clinical assessment of health technologies across Member States. Although it does not specifically regulate newborn screening programs, its principles are relevant to evidence-based evaluation of new screening technologies and interventions. By embedding the SUSP within these international agendas, Spain not only addresses domestic disparities but also contributes to the global movement toward more equitable and harmonized newborn screening systems [10].

Beyond defining the disorders included in newborn screening, the SUSP also provides standardized recommendations for biochemical and molecular confirmatory testing, thereby supporting consistent post-screening diagnostic evaluation and reducing variability in patient management.

The success of the SUSP will ultimately depend on its effective implementation at the national level. Comprehensive surveys conducted across all Spanish newborn screening centers provided detailed insights into current practices, challenges, and priorities, and were instrumental in shaping the final proposal. This consensus has been submitted to the Ministry of Health with the objective of standardizing screening offerings across ACs [8]. The establishment of a permanent multidisciplinary national advisory board is essential to oversee the future incorporation of additional disorders into the SUSP through a transparent and standardized evaluation process. Candidate conditions should be assessed according to predefined criteria, including evidence of clinical benefit, technical feasibility, analytical performance of the screening test, availability of validated biomarkers, successful pilot implementation in the Spanish newborn screening laboratories, and the existence of standardized biochemical and genetic confirmatory diagnostic pathways. This evidence-based approach will ensure that future updates to the SUSP remain scientifically robust, technically feasible, and nationally harmonized.

National guidelines should ensure comparable quality standards, validated methodologies, harmonized reporting, and equivalent clinical pathways across autonomous communities, while allowing technically validated local implementation, following the example of Italy’s centralized coordination through the Istituto Superiore di Sanità (ISS) and regional laboratories [4,5].

The SUSP should therefore be considered a dynamic framework rather than a static disease list, allowing periodic evidence-based updates as scientific knowledge and screening technologies evolve.

The establishment of a national newborn screening registry will be essential to support harmonized implementation of the SUSP. The registry should collect standardized information on screened newborns, confirmed diagnoses, biochemical and molecular confirmatory results, genotype–phenotype correlations where available, clinical outcomes, and key programme performance indicators. Such a centralized platform will facilitate quality assurance, epidemiological surveillance, benchmarking among screening centres, and future research collaborations, while providing the evidence required for periodic evaluation and SUSP updating. Long-term sustainability depends on adequate governmental funding, not only for laboratory capacity but also for workforce training and public awareness initiatives [5]. Italy’s national archive provides a valuable model for this approach [4]. The registry would also facilitate data interoperability and future joint clinical assessments across EU Member States, in line with the objectives of the EU HTA framework [10].

### Strengths and Limitations

Major strengths of this work include the participation of all newborn screening centers in Spain, ensuring that the consensus reflects real-world laboratory practice, together with the harmonization of disease nomenclature, standardized biochemical and genetic confirmatory diagnostic pathways, and the integration of biomarker-based screening entries with the counting of unique clinical condition. Collectively, these features enhance methodological transparency, reproducibility, and clinical applicability.

Nevertheless, the present consensus is intentionally limited to disorders for which validated screening biomarkers with adequate analytical performance are currently implemented in Spanish newborn screening laboratories. Consequently, some clinically relevant conditions remain excluded until sufficient evidence, validated pilot studies, and appropriate screening methodologies become available. Furthermore, successful nationwide implementation will depend on sustained political commitment, adequate resources, and long-term institutional support.

## 5. Conclusions

The SUSP represents a major milestone in the harmonization of newborn screening in Spain. Rather than being merely a list of screened diseases, it constitutes a comprehensive diagnostic framework that integrates standardized disease nomenclature, biomarkers, confirmatory biochemical investigations, and molecular diagnostic recommendations. By defining a standardized set of conditions together with harmonized diagnostic pathways, the SUSP provides a robust framework to promote equitable access to early diagnosis, confirmatory testing, and appropriate treatment across Spain, while offering a reproducible model for other countries seeking to harmonize newborn screening within decentralized healthcare systems.

Its long-term success will depend on sustained political commitment, adequate resource allocation, and adherence to ethical principles, including informed parental consent, data protection, and equitable access across regions and socioeconomic groups. A working group at the Spanish Ministry of Health is currently evaluating the proposed panel and nomenclature. If formally adopted, the SUSP has the potential to harmonize newborn screening programs across Spain and contribute to ongoing European harmonization initiatives.

Beyond its national relevance, the SUSP provides a scalable, reproducible, and consensus-based framework that may serve as a model for harmonizing newborn screening programs in other decentralized healthcare systems, while informing future international efforts toward standardized newborn screening policies.

## Figures and Tables

**Table 1:** Main Wilson and Jungner criteria considered for the designation of primary conditions in the Spanish Uniform Screening Panel (SUSP).

Criterion	Definition	Required Evidence
Net Health Benefit	Evidence demonstrating that newborn screening improves clinically relevant outcomes compared with usual clinical diagnosis by enabling earlier detection, timely treatment, and improved long-term prognosis.	Studies demonstrating reduced morbidity and mortality in patients diagnosed early [7].
Technical Feasibility	Availability of reliable, validated, and reproducible screening methods with adequate analytical performance (including sensitivity, specificity, and overall test accuracy) suitable for implementation in routine newborn screening laboratories.	Biomarkers or methods already validated in Spanish screening laboratories [6].
Treatment Accessibility	Existence of effective therapies or interventions that can reduce morbidity or mortality associated with the disorder.	Treatments available in Spain and accessible at the national level [5].

**Table 2:** Amino acid disorders: primary and secondary conditions (PC and SC) in the Spanish Uniform Screening Panel (SUSP), grouped by primary marker detected by newborn screening.

First-Tier Screening Biomarker (DBS)	Official Condition or Group of Conditions (Spanish Ministry of Health)	Condition	ORPHA Code	Consensus 1: PC 2: SC	OMIM Disease Number	Associated Disease Gene(s) *	OMIM Gene Number	Recommended Biochemical or Functional Confirmatory Tests	Recommended Gene Panel for Differential Molecular Diagnosis **	RUSP 1: PC 2: SC	Portugal 1: PC 2: SC
Arg ↑	Argininemia	Argininemia	90	2	207800	*ARG1*	608313	P-AA, U-AA	*ARG1*	2	1
Cit ↑	Argininosuccinic aciduria	Argininosuccinic aciduria (+ASA)	23	1	207900	*ASL*	608310	P-AA, U-AA, U-AO, urine galactose	*ASS1/ASL/* *DLD/* *PC/* *SLC25A13/* *SLC7A7*	1	1
	Citrullinemia type I	Citrullinemia type I	247525	1	215700	*ASS1*	603470			1	1
	Citrullinemia type II (citrin deficiency)	Citrullinemia type II (citrin deficiency)	247585	2	605814, 603471	*SLC25A13*	603859			2	
	Pyruvate carboxylase deficiency	Pyruvate carboxylase deficiency	3008	2	266150	*PC*	608786			N.I.	N.I.
	Dihydrolipoamide dehydrogenase deficiency	Dihydrolipoamide dehydrogenase deficiency	2394	2	246900	*DLD*	238331			N.I.	N.I.
	Lysinuric protein intolerance	Lysinuric protein intolerance	470	2	222700	*SLC7A7*	603593			N.I.	N.I.
Cit ↓ and/or C5OH ↑	Leigh syndrome due to MT-ATP6 mutations	Leigh syndrome due to MT-ATP6 mutations	320360	2	551500	*MT-ATP6*	516060	P-AA, U-AA, U-AO, urine orotic acid	*CA5A/* *CPS1/* *MT-ATP6/* *NAGS/OTC*	N.I.	N.I.
	Carbonic anhydrase VA deficiency	Carbonic anhydrase VA deficiency	401948	2	615751	*CA5A*	114761			N.I.	N.I.
Gln ↑ and/or Cit ↓	Carbamoyl phosphate synthetase I deficiency	Carbamoyl phosphate synthetase I deficiency	147	2	237300	*CPS1*	608307	P-AA, U-AA, U-AO, urine orotic acid	*CAD* *CA5A/* *CPS1/* *MT-ATP6/* *NAGS/* *OTC*	N.I.	N.I.
	N-acetylglutamate synthase deficiency	N-acetylglutamate synthase deficiency	927	2	237310	*NAGS*	608300			N.I.	N.I.
	Ornithine transcarbamylase deficiency	Ornithine transcarbamylase deficiency	664	2	311250	*OTC*	300461			N.I.	N.I.
XLeu, Val, XLeu/Phe ↑	Maple syrup urine disease #	Maple syrup urine disease, E1α subunit deficiency	511	1	248600	*BCKDHA*	608348	P-AA, U-AA, U-AO	*BCKDHA/* *BCKDHB/* *BCAT2* *DBT/* *DLD/* *PPM1K*	1	1
		Maple syrup urine disease, E1β subunit deficiency	511	1	620698	*BCKDHB*	248611			1	1
		Maple syrup urine disease, E2 subunit deficiency	511	1	620699	*DBT*	248610			1	1
		Maple syrup urine disease, dihydrolipoamide dehydrogenase deficiency	2395	1	246900	*DLD*	238331			1	1
		Maple syrup urine disease, PPM1K deficiency	248162	1	615135	*PPM1K*	611065			1	1
	Hypervalinemia and hyperleucine-isoleucinemia	Hypervalinemia and hyperleucine-isoleucinemia	None	2	618850	*BCAT2*	113530			N.I.	N.I.
XLeu, Val ↓	Branched-chain α-ketoacid dehydrogenase kinase deficiency	Branched-chain α-ketoacid dehydrogenase kinase deficiency	308410	2	614923	*BCKDK*	614901	P-AA, U-AA	*BCKDK*	N.I.	N.I.
Met, Met/Phe ↑	Classical homocystinuria #	Classical homocystinuria due to cystathionine beta-synthase deficiency	394	1	236200	*CBS*	613381	P-AA, U-AA, total plasma homocysteine, plasma S-adenosyl-methionine and S-adenosyl-homocysteine	*ADK/* *AHCY/* *CBS/* *GNMT/* *MAT1A*	1	1
	Hypermethioninemia disorders	Hypermethioninemia due to methionine adenosyltransferase I/III deficiency	168598	2	250850	*MAT1A*	610550			2	1
		Hypermethioninemia due to glycine N-methyltransferase deficiency	289891	2	606664	*GNMT*	606628			2	2
		Hypermethioninemia due to adenosylhomocysteinase deficiency	88618	2	613752	*AHCY*	180960			2	2
		Hypermethioninemia due to adenosine kinase deficiency	289290	2	614300	*ADK*	102750			2	2
Met, Met/Phe ↓	Non-classical homocystinuria	Homocystinuria due to methylenetetrahydrofolate reductase deficiency (MTHFR deficiency)	395	2	236250	*MTHFR*	607093	P-AA, U-AA, U-AO, total plasma homocysteine	*MTHFR/* *MTR/* *MTRR*	1	2
		Homocystinuria-megaloblastic anemia, cblE type	2169	2	236270	*MTRR*	602568			1	2
		Homocystinuria-megaloblastic anemia, cblG type	2170	2	250940	*MTR*	156570			1	2
Phe, Phe/Tyr ↑	Phenylketonuria #	Phenylketonuria/hyperphenylalaninemia due to phenylalanine hydroxylase deficiency	716	1	261600	*PAH*	612349	P-AA, U-AA, U-AO, urine pterins, dihydropteridine reductase activity in dried blood spot, BH4 loading test	*DNAJC12/* *GCH1/* *PAH/* *PCBD1/* *PTS/* *QDPR*	1	1
	Hyperphenylalaninemia due to DNAJC12 deficiency	Hyperphenylalaninemia due to DNAJC12 deficiency (defect of phenylalanine, tyrosine, and tryptophan hydroxylases)	508523	2	617384	*DNAJC12*	606060			2	1
	Tetrahydrobiopterin (BH4) biosynthesis defects	GTP cyclohydrolase I deficiency	2102	2	233910	*GCH1*	600225			2	2
		6-pyruvoyl-tetrahydropterin synthase deficiency	13	2	261640	*PTS*	612719			2	2
	Tetrahydrobiopterin (BH4) regeneration defects	Pterin-4α-carbinolamine dehydratase deficiency	1578	2	264070	*PCBD1*	126090			2	2
		Dihydropteridine reductase deficiency	226	2	261630	*QDPR*	612676			2	2
SA and Tyr ↑	Tyrosinemia type I #	Tyrosinemia type I	882	1	276700	*FAH*	613871	P-AA, urine SA	*FAH/* *HPD/* *TAT*	1	1
Tyr ↑	Other tyrosinemias	Tyrosinemia type II	28378	1	276600	*TAT*	613018	P-AA, urine SA		2	1
		Tyrosinemia type III	69723	2	276710	*HPD*	609695			2	1
SA ↑	Maleylacetoacetate isomerase deficiency	Maleylacetoacetate isomerase deficiency	None	2	617596	*GSTZ1*	603758	P-AA, urine SA	*FAH/GSTZ1*	N.I.	N.I.

↑ Increased; ↓: Decreased; #: Disease included in Basic Common Portfolio in Spain. * “Associated Disease Gene(s)” identifies the causative gene(s) for each individual disorder; ** “Recommended Gene Panel for Differential Molecular Diagnosis” lists the genes that should be considered during the molecular diagnostic evaluation initiated by an abnormal primary screening biomarker, thereby facilitating the differential diagnosis of disorders with overlapping biochemical profiles; ASA: Argininosuccinic acid; Arg: Arginine; Cit: Citrulline; DBS: dried blood spot; Gln: Glutamine; Met: Methionine; Met/Phe: Ratio of methionine to phenylalanine; Met/XLeu: Ratio of methionine to branched-chain amino acids. N.I.: not included; P-AA: plasma amino acids; Phe: Phenylalanine; Phe/Tyr: Ratio pf phenylalanine to tyrosine; PC: primary condition; RUSP: Recommended Uniform Screening Panel from the U.S. SA: Succinylacetone. SC: Secondary condition; Tyr: Tyrosine; U-AA: urinary amino acids; U-AO: urinary organic acids; Val: Valine; XLeu: Branched-chain amino acids (leucine, isoleucine, alloisoleucine, hydroxyproline mix); XLeu/Phe: Ratio of branched-chain amino acids to phenylalanine.

**Table 3:** Organic acid disorders: Primary and secondary conditions (PC and SC) in the Spanish Uniform Screening Panel (SUSP), grouped by primary marker detected by newborn screening.

First-Tier Screening Biomarker (DBS)	Official Condition or Group of Conditions (Spanish Ministry of Health)	Condition	ORPHA Code	Consensus 1: PC 2: SC	OMIM Disease Number	Associated Disease Gene(s) *	OMIM Gene Number	Recommended Biochemical or Functional Confirmatory Tests	Recommended Gene Panel for Differential Molecular Diagnosis **	RUSP 1: PC 2: SC	Portugal 1: PC 2: SC
C3, C3/C2 ↑	Propionic acidemia #	Propionic acidemia due to propionyl-CoA carboxylase alpha subunit deficiency	35	1	606054	*PCCA*	232000	U-AO, P-AA, U-AA, P-ACN, U-ACN, total plasma homocysteine	*ABCD4/* *ACSF3/* *ALDH6A1/* *AMN/* *CA5A/* *CBLIF/* *CD320/* *CUBN/* *HCFC1/* *LMBRD1/* *MMAA/* *MMAB/* *MMACHC/* *MMADHC/* *MCEE/* *MLYCD/* *MMUT/* *PCCA/* *PCCB/* *PRDX1/* *TCN2/THAP11/* *ZNF143*	1	1
		Propionic acidemia due to propionyl-CoA carboxylase beta subunit deficiency	35	1	606054	*PCCB*	232050			1	1
	Methylmalonic acidemia #	Methylmalonic acidemia due to methylmalonyl-CoA mutase deficiency, (±C4DC ↑)	27	1	251000	*MMUT*	609058			1	1
		Vitamin B12-responsive methylmalonic acidemia, cblA type, (±C4DC ↑)	79310	1	251100	*MMAA*	607481			1	1
		Vitamin B12-responsive methylmalonic acidemia, cblB type, (±C4DC ↑)	79311	1	251110	*MMAB*	607568			1	1
		Methylmalonic acidemia due to methylmalonyl-CoA epimerase deficiency	308425	2	251120	*MCEE*	608419			N.I.	1
		Methylmalonic semialdehyde dehydrogenase deficiency	289307	2	614105	*ALDH6A1*	603178			N.I.	2
		Combined malonic and methylmalonic aciduria (±C3DC ↑)	289504	2	614265	*ACSF3*	614245			N.I.	2
	Methylmalonic acidemia with homocystinuria #	Methylmalonic acidemia with homocystinuria, cblC type (± Met ↓)	79282	1	277400	*MMACHC/* *PRDX1*	609831/ 176763			2	1
		Methylmalonic acidemia with or without homocystinuria, cblD type (± Met ↓)	79283	1	277410	*MMADHC*	611935			2	1
		Methylmalonic acidemia with homocystinuria, cblF type (± Met ↓)	79284	1	277380	*LMBRD1*	612625			2	1
		Methylmalonic acidemia with homocystinuria, cblJ type (± Met ↓)	369955	1	614857	*ABCD4*	603214			2	1
		Methylmalonic acidemia with homocystinuria, cblX type (± Met ↓)	369962	1	309541	*HCFC1*	300019			2	1
		Methylmalonic acidemia due to intrinsic factor deficiency (± Met ↓)	332	1	261000	*CBLIF*	609342			2	1
		Imerslund–Gräsbeck syndrome type 1 (± Met ↓)	35858	1	261100	*CUBN*	602997			2	1
		Imerslund–Gräsbeck syndrome type 2 (±Met ↓)	35858	1	618882	*AMN*	605799			2	1
		Transient methylmalonic acidemia due to transcobalamin receptor defect (±Met ↓)	280183	1	613646	*CD320*	606475			2	1
		Methylmalonic acidemia due to transcobalamin II deficiency (±Met ↓)	859	1	275350	*TCN2*	613441			2	1
		Methylmalonic acidemia with homocystinuria, cblL type	None	2	620940	*THAP11*	609119			N.I.	N.I.
		Methylmalonic acidemia with homocystinuria due to ZNF143 deficiency	None	2	None	*ZNF143*	603433			N.I.	N.I.
	Malonic aciduria	Malonic aciduria (±C3DC ↑)	943	2	248360	*MLYCD*	606761			2	1
	Carbonic anhydrase VA deficiency	Carbonic anhydrase VA deficiency (±C5OH ↑, Cit ↓)	401948	2	615751	*CA5A*	114761			N.I.	N.I.
C4, C4/C2 ↑	Short-chain acyl-CoA dehydrogenase deficiency	Short-chain acyl-CoA dehydrogenase deficiency (SCAD deficiency)	26792	2	201470	*ACADS*	606885	U-AO, P-ACN, U-ACN	*ACAD8/* *ACADS/* *ECHDC1/* *ETHE1*	2	N.I.
	Isobutyrylglycinuria	Isobutyrylglycinuria	79159	2	611283	*ACAD8*	604773			2	N.I.
C4DC ↑	Encephalomyopathy with methylmalonic aciduria due to succinyl-CoA ligase deficiency	Mitochondrial DNA depletion syndrome (encephalomyopathy with methylmalonic aciduria) due to succinyl-CoA ligase alpha subunit deficiency	17	2	245400	*SUCLG1*	611224	U-AO, P-ACN, U-ACN	*SUCLA2/* *SUCLG1*	N.I.	N.I.
		Mitochondrial DNA depletion syndrome (encephalomyopathy with methylmalonic aciduria) due to succinyl-CoA ligase beta subunit deficiency	1933	2	612073	*SUCLA2*	603921			N.I.	N.I.
C4 + FIGLU ↑	Formiminoglutamic aciduria	Formiminoglutamic aciduria	51208	2	229100	*FTCD*	606806	U-AO, P-ACN, U-ACN	*FTCD*	N.I.	N.I.
C4 + C5 ↑	Ethylmalonic aciduria due to mitochondrial sulfur dioxygenase deficiency	Ethylmalonic aciduria due to mitochondrial sulfur dioxygenase deficiency	51188	2	602473	*ETHE1*	608451	U-AO, P-ACN, U-ACN	*ETHE1*	N.I.	2
C4-OH ↑	3-hydroxyisobutyric aciduria	3-hydroxyisobutyric aciduria	88639	2	250620	*HIBCH*	610690	U-AO, P-ACN, U-ACN	*HADH/HIBCH*	N.I.	2
	Short-chain 3-hydroxyacyl-CoA dehydrogenase deficiency	Short-chain 3-hydroxyacyl-CoA dehydrogenase deficiency (SCHAD deficiency)	71212	2	231530	*HADH*	601609			2	1
C5, C5/C0 ↑	Isovaleric acidemia #	Isovaleric acidemia	33	1	243500	*IVD*	607036	U-AO, P-ACN, U-ACN, P-AA, U-AA	*ACADSB/IVD*	1	1
	2-methylbutyrylglycinuria	2-methylbutyrylglycinuria	79157	2	610006	*ACADSB*	600301			2	2
C5DC, C5DC/C3DC ↑	Glutaric aciduria type I #	Glutaric aciduria type I	25	1	231670	*GCDH*	608801	U-AO, P-ACN, U-ACN	*GCDH/SUGCT*	1	1
	Glutaric aciduria type III	Glutaric aciduria type III	35706	2	231690	*SUGCT*	609187			N.I.	2
C5OH + C6DC ↑	3-hydroxy-3-methylglutaric aciduria #	3-hydroxy-3-methylglutaric aciduria	20	1	246450	*HMGCL*	613898	U-AO, P-ACN, U-ACN	*AUH/HMGCL*	1	1
C5OH ↑	Beta-ketothiolase deficiency #	Beta-ketothiolase deficiency (±C4OH y C5:1 ↑)	134	1	203750	*ACAT1*	607809	U-AO, P-ACN, U-ACN, serum biotinidase activity	*ACAT1/* *AUH/BTD/* *CA5A/* *HLCS/* *HMGCL/* *HSD17B10/* *MCCC1/* *MCCC2*	1	2
	Biotinidase deficiency #	Biotinidase deficiency (±C3 ↑)	79241	1	253260	*BTD*	609019			1	N.I.
	3-methylglutaconic aciduria type I	3-methylglutaconic aciduria type I	67046	2	250950	*AUH*	600529			2	2
	3-methylcrotonylglycinuria	3-methylcrotonylglycinuria type I (± C5:1 ↑)	6	2	210200	*MCCC1*	609010			1	1
		3-methylcrotonylglycinuria type II (± C5:1 ↑)	6	2	210210	*MCCC2*	609014			1	1
	Holocarboxylase synthetase deficiency	Holocarboxylase synthetase deficiency (C3 ↑)	79242	2	253270	*HLCS*	609018			1	2
	2-methyl-3-hydroxybutyric aciduria	2-methyl-3-hydroxybutyric aciduria	391417	2	300438	*HSD17B10*	300256				
	Carbonic anhydrase VA deficiency	Carbonic anhydrase VA deficiency (±C3 ↑ y Cit ↓)	401948	2	615751	*CA5A*	114761			2	N.I.
C6DC ↑	Other 3-methylglutaconic aciduria disorders	Barth syndrome	111	2	302060	*TAFAZZIN*	300394	U-AO, P-ACN, U-ACN, P-AA	*AGK/* *ATP5F1A/* *ATP5F1D/* *ATP5F1E/* *ATP5MD/* *ATPAF2/* *CLPB/* *DNAJC19/* *HTRA2/* *MICOS13/* *OPA3/* *SERAC1/* *TAFAZZIN/* *TIMM50/* *TMEM70*	2	N.I.
		3-methylglutaconic aciduria with deafness–encephalopathy–Leigh-like syndrome (MEGDEL syndrome)	352328	2	614739	*SERAC1*	614725			2	N.I.
		3-methylglutaconic aciduria type III	67047	2	258501	*OPA3*	606580			2	N.I.
		Combined oxidative phosphorylation deficiency 37	None	2	618329	*MICOS13*	616658			2	N.I.
		3-methylglutaconic aciduria type V	66634	2	610198	*DNAJC19*	608977			2	N.I.
		3-methylglutaconic aciduria type VII	445038	2	619835/ 616271	*CLPB*	616254			2	N.I.
		3-methylglutaconic aciduria type VIII	505208	2	617248	*HTRA2*	606441			2	N.I.
		3-methylglutaconic aciduria type IV	505216	2	617698	*TIMM50*	607381			2	N.I.
		Mitochondrial complex V (ATP synthase) deficiency, nuclear type I	254913	2	604273	*ATPAF2*	608918			2	N.I.
		Mitochondrial complex V (ATP synthase) deficiency, nuclear type II	1194	2	614052	*TMEM70*	612418			2	N.I.
		Mitochondrial complex V (ATP synthase) deficiency, nuclear type III	254913	2	614053	*ATP5F1E*	606153			2	N.I.
		Mitochondrial complex V (ATP synthase) deficiency, nuclear type IVB	254913	2	615228	*ATP5F1A*	164360			2	N.I.
		Mitochondrial complex V (ATP synthase) deficiency, nuclear type V	254913	2	618120	*ATP5F1D*	603150			2	N.I.
		Mitochondrial complex V (ATP synthase) deficiency, nuclear type VI	254913	2	618683	*ATP5MD*	615204			2	N.I.
		Sengers syndrome	1369	2	212350	*AGK*	610345			2	N.I.
Biotinidase activity ↓	Biotinidase deficiency #	Biotinidase deficiency	79241	1	253260	*BTD*	609019	Serum biotinidase activity, P-ACN, U-ACN, U-AO	*BTD*	1	N.I.

↑ Increased; ↓: Decreased; #: Disease included in Basic Common Portfolio in Spain; * “Associated Disease Gene(s)” identifies the causative gene(s) for each individual disorder; ** “Recommended Gene Panel for Differential Molecular Diagnosis” lists the genes that should be considered during the molecular diagnostic evaluation initiated by an abnormal primary screening biomarker, thereby facilitating the differential diagnosis of disorders with overlapping biochemical profiles; C3: Propionylcarnitine; C3/C2: Ratio of propionylcarnitine to acetylcarnitine; C3DC: Methylmalonylcarnitine; C4DC: Succinylcarnitine; C4OH: Hydroxybutyrylcarnitine; C5: Isovalerylcarnitine; C5/C0: Ratio of isovalerylcarnitine to free carnitine; C5:1: Tiglylcarnitine; C5DC: Glutarylcarnitine; C5DC/C3DC: Ratio of glutarylcarnitine to methylmalonylcarnitine; C5OH: 3-Hydroxyisovalerylcarnitine; C6DC: Adipylcarnitine and 3-methylglutyrilcarnitine; DBS: dried blood spot; FIGLU: Formiminoglutamic acid; N.I.: not included; P-AA: plasma amino acids; P-ACN: plasma acylcarnitines; PC: primary condition; RUSP: Recommended Uniform Screening Panel from the U.S.; U-AA: urinary amino acids; U-ACN: urinary acylcarnitines; U-AO: urinary organic acids.

**Table 4:** Mitochondrial fatty acid β-oxidation disorders: Primary and secondary conditions (PC and SC) in the Spanish Uniform Screening Panel (SUSP), grouped by primary marker detected by newborn screening.

First-Tier Screening Biomarker (DBS)	Official Condition or Group of Conditions (Spanish Ministry of Health)	Condition	ORPHA Code	Consensus 1: PC 2: SC	OMIM Disease Number	Associated Disease Gene(s) *	OMIM Gene Number	Recommended Biochemical or Functional Confirmatory Tests	Recommended Gene Panel for Differential Molecular Diagnosis **	RUSP 1: PC 2: SC	Portugal 1: PC 2: SC
C0 ↓ and other acylcarnitines ↓	Primary carnitine deficiency (carnitine transporter deficiency) #	Primary carnitine deficiency (carnitine transporter deficiency, CTD)	158	1	212140	*SLC22A5*	603377	P-ACN, plasma free and total carnitine	*SLC22A5*	1	1
C0, C0/(C16 + C18) ↑ and long-chain acylcarnitines ↓	Carnitine palmitoyltransferase I deficiency	Carnitine palmitoyltransferase I deficiency (CPT1 deficiency)	156	1	255120	*CPT1A*	600528	P-ACN, plasma free and total carnitine, DBS-ACN and free carnitine, U-AO	*COASY/* *CPT1A/* *PANK1/* *PANK2/* *PANK3/* *PANK4/* *PPCDC/* *PPCS*	2	1
	Coenzyme A synthase deficiency	Neurodegeneration with brain iron accumulation type 6 (NBIA type 6)	397725	2	615643	*COASY*	609855			N.I.	2
		Dilated cardiomyopathy type 2C	154	2	618189	*PPCS*	609853			N.I.	2
		Phosphopantothenoylcysteine decarboxylase deficiency	None	2	None	*PPCDC*	609854			N.I.	2
		Pantothenate kinase deficiency type 1 (PANK1 deficiency)	None	2	None	*PANK1*	606160			N.I.	2
		Pantothenate kinase deficiency type 2 (PANK2 deficiency)	157850	2	234200	*PANK2*	606157			N.I.	2
		Pantothenate kinase deficiency type 3 (PANK3 deficiency)	None	2	None	*PANK3*	606161			N.I.	2
		Pantothenate kinase deficiency type 4 (PANK4 deficiency)	98993	2	619593	*PANK4*	606162			N.I.	2
C6, C8, C8/C2, C8/C10 ↑	Medium-chain acyl-CoA dehydrogenase deficiency #	Medium-chain acyl-CoA dehydrogenase deficiency (MCAD deficiency)	42	1	201450	*ACADM*	607008	P-ACN, U-AO	*ACADM*	1	1
C14:1, C14:2, C14, C14:1/C2 ↑	Very long-chain acyl-CoA dehydrogenase deficiency #	Very long-chain acyl-CoA dehydrogenase deficiency (VLCAD deficiency)	26793	1	201475	*ACADVL*	609575	P-ACN, U-AO	*ACADVL*	1	1
C16-OH, C18-OH, C14:1, C16:1, C18:1, C16OH/C16 ↑	Long-chain 3-hydroxyacyl-CoA dehydrogenase deficiency #	Long-chain 3-hydroxyacyl-CoA dehydrogenase deficiency (LCHAD deficiency)	5	1	609016	*HADHA*	600890	P-ACN, U-AO	*HADHA/* *HADHB*	1	1
	Mitochondrial trifunctional protein deficiency type II	Mitochondrial trifunctional protein deficiency type II (MTP deficiency)	746	1	620300 609015	*HADHB*	143450			1	1
(C16 + C18:1)/C2, C16, C18, C16:1, C18:1 ↑ and C0/(C16 + C18) ↓	Carnitine palmitoyltransferase II deficiency	Carnitine palmitoyltransferase II deficiency (CPT2 deficiency)	157	1	600649/ 608836/ 255110	*CPT2*	600650	P-ACN, U-AO	*CPT2/* *SLC25A20*	2	1
	Carnitine-acylcarnitine translocase deficiency	Carnitine-acylcarnitine translocase deficiency (CACT deficiency)	159	1	212138	*SLC25A20*	613698			2	1
Several short-, medium-, long-acylcarnitine species ↑	Multiple acyl-CoA dehydrogenase deficiency	Multiple acyl-CoA dehydrogenase deficiency (MADD, glutaric acidemia type II) due to ETFDH deficiency	26791	1	231680	*ETFDH*	231675	P-ACN, U-AO	*ETFA/* *ETFB/* *ETFDH/* *FLAD1/* *SLC25A32/* *SLC52A1/* *SLC52A2/* *SLC52A3*	2	1
		Multiple acyl-CoA dehydrogenase deficiency (MADD, glutaric acidemia type II) due to ETFA deficiency	26791	1	231680	*ETFA*	608053			2	1
		Multiple acyl-CoA dehydrogenase deficiency (MADD, glutaric acidemia type II) due to ETFB deficiency	26791	1	231680	*ETFB*	130410			2	1
		FAD synthase deficiency	26791	2	255100	*FLAD1*	610595			2	1
		Riboflavin transporter deficiency	411712	2	615026	*SLC52A1*	607883			2	1
		Riboflavin transporter deficiency type II (Brown–Vialetto–Van Laere syndrome 2)	97229	2	614707	*SLC52A2*	607882			2	1
		Brown–Vialetto–Van Laere syndrome 1	97229	2	211530	*SLC52A3*	613350			2	1
		Folate transporter deficiency	26791	2	616839	*SLC25A32*	138480			2	1
C10:2 ↑	2,4-dienoyl-CoA reductase deficiency	2,4-dienoyl-CoA reductase deficiency	None	2	None	*DECR1*	222745	P-ACN, P-AA, U-AA	*DECR1/* *NADK2*	2	2
		Mitochondrial NAD kinase 2 deficiency (NADK2 deficiency)	431361	2	616034	*NADK2*	615787			2	2

↑ Increased; ↓: Decreased; #: Disease included in Basic Common Portfolio in Spain; * “Associated Disease Gene(s)” identifies the causative gene(s) for each individual disorder; ** “Recommended Gene Panel for Differential Molecular Diagnosis” lists the genes that should be considered during the molecular diagnostic evaluation initiated by an abnormal primary screening biomarker, thereby facilitating the differential diagnosis of disorders with overlapping biochemical profiles; C0: Free carnitine; C0/(C16 + C18): Ratio of free carnitine to palmitoylcarnitine plus Stearoylcarnitine; C2: Acetylcarnitine; C6: Hexanoylcarnitine; C8: Octanoylcarnitine; C8/C2: Ratio of octanoylcarnitine to acetylcarnitine; C8/C10: Ratio of octanoylcarnitine to decanoylcarnitine; C10:2: Decadienoylcarnitine; C14: Tetradecanoylcarnitine; C14:1: Tetradecenoylcarnitine; C14:2: Tetradecadienoylcarnitine; C14:1/C2: Ratio of tetradecenoylcarnitine to acetylcarnitine; C16: Palmitoylcarnitine; (C16 + C18:1)/C2: Ratio of palmitoylcarnitine plus oleoylcarnitine to acetylcarnitine; C16:1: Palmitoleoylcarnitine; C16-OH: Hydroxypalmitoylcarnitine; C16OH/C16: Ratio of hydroxypalmitoylcarnitine to palmitoylcarnitine; C18: Stearoylcarnitine; C18:1: Oleoylcarnitine; C18-OH: Hydroxystearoylcarnitine; DBS: dried blood spot; N.I.: not included; P-AA: plasma amino acids; P-ACN: plasma acylcarnitines; PC: primary condition; RUSP: Recommended Uniform Screening Panel from the U.S.; SC: Secondary condition; U-AA: urinary amino acids; U-AO: urinary organic acids.

**Table 5:** Other inherited metabolic disorders: Primary and secondary conditions (PC and SC) in the Spanish Uniform Screening Panel (SUSP), grouped by primary marker detected by newborn screening.

First-Tier Screening Biomarker (DBS)	Official Condition or Group of Conditions (Spanish Ministry of Health)	Condition	ORPHA Code	Consensus 1: PC 2: SC	OMIM Disease Number	Associated Disease Gene(s) *	OMIM Gene Number	Recommended Biochemical or Functional Confirmatory Tests	Recommended Gene Panel for Differential Molecular Diagnosis **	RUSP 1: PC 2: SC	Portugal 1: PC 2: SC
C26:0-LPC ↑ and ±C24:LPC, C26, C24, C26/C22, C24/C22 ↑	X-linked adrenoleuko-dystrophy #	X-linked adrenoleukodystrophy (X-ALD)	43	1	300100	*ABCD1*	300371	Plasma very long-chain fatty acids, LPC-C24:0, LPC-C26:0, erythrocyte plasmalogens (phytanic and pristanic acids may not be informative in the neonatal period)	*ABCD1/* *ACOX1/* *HSD17B4/* *PEX1/* *PEX10/* *PEX11B/* *PEX12/* *PEX13/* *PEX14/* *PEX16/* *PEX19/* *PEX2/* *PEX26/* *PEX3/* *PEX5/* *PEX6*	1	N.I.
	Espectro Zelweger	Peroxisome biogenesis disorder 1A	912	2	214100	*PEX1*	602136			N.I.	N.I.
		Peroxisome biogenesis disorder 2A	912	2	214110	*PEX5*	600414			N.I.	N.I.
		Peroxisome biogenesis disorder 3A	912	2	614859	*PEX12*	601758			N.I.	N.I.
		Peroxisome biogenesis disorder 4A	912	2	614862	*PEX6*	601498			N.I.	N.I.
		Peroxisome biogenesis disorder 5A	912	2	614866	*PEX2*	170993			N.I.	N.I.
		Peroxisome biogenesis disorder 6A	912	2	614870	*PEX10*	602859			N.I.	N.I.
		Peroxisome biogenesis disorder 7A	912	2	614872	*PEX26*	608666			N.I.	N.I.
		Peroxisome biogenesis disorder 8A	912	2	614876	*PEX16*	603360			N.I.	N.I.
		Peroxisome biogenesis disorder 10	912	2	614882/617370	*PEX3*	603164			N.I.	N.I.
		Peroxisome biogenesis disorder 11A	912	2	614883	*PEX13*	601789			N.I.	N.I.
		Peroxisome biogenesis disorder 12A	912	2	614886	*PEX19*	600279			N.I.	N.I.
		Peroxisome biogenesis disorder 13A	912	2	614887	*PEX14*	601791			N.I.	N.I.
		Peroxisome biogenesis disorder 14B	912	2	614920	*PEX11B*	603867			N.I.	N.I.
	Peroxisomal fatty acid beta-oxidation defect	Peroxisomal acyl-CoA oxidase deficiency	2971	2	264470	*ACOX1*	609751			N.I.	N.I.
		Peroxisomal D-bifunctional protein deficiency	300	2	261515	*HSD17B4*	601860			N.I.	N.I.
Lysosomal alpha-glucosidase activity ↓	Pompe disease	Pompe disease	365	1	232300	*GAA*	606800	Lysosomal alpha-glucosidase activity in lymphocytes, urine oligosaccharides	*GAA*	1	N.I.
GAL or GAL-1P ↑	Classic galactosemia	Classic galactosemia due to galactose-1-phosphate uridyltransferase deficiency	79239	1	230400	*GALT*	606999	Plasma galactose (prior to dietary intake), erythrocyte galactose-1-phosphate, plasma galactitol	*GALE/* *GALK1/* *GALT/* *GALM*	1	N.I.
	Other galactosemias	Galactosemia due to UDP-galactose epimerase deficiency	79238	2	230350	*GALE*	606953			2	N.I.
		Galactosemia due to galactose mutarotase deficiency	570422	2	618881	*GALM*	618881			N.I.	N.I.
		Galactosemia due to galactokinase deficiency	79237	2	230200	*GALK1*	604313			2	N.I.
Alpha-L-iduronidase activity ↓	Mucopolysaccharidosis type I (MPS I)	Mucopolysaccharidosis type I (MPS I)	579	1	607014/607015/607016	*IDUA*	252800	Alpha-L-iduronidase activity in leukocytes, urine glycosaminoglycans	*IDUA*	1	N.I.

↑ Increased; ↓: Decreased; #: Disease included in Basic Common Portfolio in Spain; * “Associated Disease Gene(s)” identifies the causative gene(s) for each individual disorder; ** “Recommended Gene Panel for Differential Molecular Diagnosis” lists the genes that should be considered during the molecular diagnostic evaluation initiated by an abnormal primary screening biomarker, thereby facilitating the differential diagnosis of disorders with overlapping biochemical profiles; C22: Docosanoylcarnitine; C24: Tetracosanoylcarnitine; C24/C22: Ratio of tetracosanoylcarnitine to docosanoylcarnitine; C26: Hexacosanoylcarnitine; C26/C22: Ratio of hexacosanoylcarnitine to docosanoylcarnitine; C24:0-LPC: Lysophosphatidylcholine with 24-carbon saturated fatty acid; C26:0-LPC: Lysophosphatidylcholine with 26-carbon saturated fatty acid; DBS: dried blood spot; Gal: Galactose; Gal-1P: Galactose-1-phosphate. N.I.: not included; PC: primary condition; RUSP: Recommended Uniform Screening Panel from the U.S.; SC: Secondary condition.

**Table 6:** Endocrine disorders: Primary and secondary conditions (PC and SC) in the Spanish Uniform Screening Panel (SUSP), grouped by primary marker detected by newborn screening.

First-Tier Screening Biomarker (DBS)	Official Condition or Group of Conditions (Spanish Ministry of Health	Condition	ORPHA Code	Consensus 1: PC 2: SC	OMIM Disease Number	Associated Disease Gene(s) *	OMIM Gene Number	Recommended Biochemical or Functional Confirmatory Tests	Recommended Gene Panel for Differential Molecular Diagnosis **	RUSP 1: PC 2: SC	Portugal 1: PC 2: SC
17OH progesterone ↑	Congenital adrenal hyperplasia disorders #	21-hydroxylase deficiency	90794	1	201910	*CYP21A2*	613815	Plasma steroid profile	*CYP11B1/* *CYP17A1/* *CYP21A2/* *HSD3B2/* *POR/* *STAR*	1	N.I.
		Lipoid congenital adrenal hyperplasia due to steroidogenic acute regulatory protein deficiency	90790	1	201710	*STAR*	600617			1	N.I.
		Congenital adrenal hyperplasia due to 3-beta-hydroxysteroid dehydrogenase deficiency	90791	1	201810	*HSD3B2*	613890			1	N.I.
		Congenital adrenal hyperplasia due to 11-beta-hydroxylase deficiency	90795	1	202010	*CYP11B1*	610613			1	N.I.
		Congenital adrenal hyperplasia due to 17-alpha-hydroxylase deficiency	90793	1	202110	*CYP17A1*	609300			1	N.I.
		Congenital adrenal hyperplasia due to cytochrome P450 oxidoreductase deficiency	95699	1	613571	*POR*	124015			1	N.I.
TSH ↑ or T4 ↓	Congenital hypothyroidism #	Congenital hypothyroidism due to TSH receptor mutations	90673	1	275200	*TSHR*	603372	Thyroid-stimulating hormone (TSH) and free thyroxine (fT4) or total thyroxine (T4) in venous blood	*DIO1/* *DUOX2/* *DUOXA2/* *FOXE1/* *GLIS3/* *GNAS/* *HESX1/* *IGSF1/* *IYD/* *LEPR/* *LHX3/* *LHX4/* *NKX2-1/* *NKX2-5/* *OTX2/* *PAX8/* *PROP1/* *PROU1F1/* *SECISBP2/* *SLC26A4/* *SLC5A5/* *SOX3/* *TBL1X/* *TG/* *TRHR/* *TRU-TCA1-1/* *TSHB/* *TSHR/* *TPO/*	1	1
		Hypothyroidism, congenital, due to thyroid dysgenesis or hypoplasia	95720	1	218700	*PAX8*	167415			1	1
		Hypothyroidism, congenital nongoitrous, 5	95720	1	225250	*NKX2-5*	600584			1	1
		Bamforth–Lazarus syndrome	1226	1	241850	*FOXE1*	602617			1	1
		Brain–lung–thyroid syndrome	209905	1	610978	*NKX2-1*	600635			1	1
		Neonatal diabetes–congenital hypothyroidism–congenital glaucoma–hepatic fibrosis–polycystic kidney syndrome	79118	1	610199	*GLIS3*	610192			1	1
		Thyroid dyshormonogenesis 3	95716	1	274700	*TG*	188450			1	1
		Thyroid dyshormonogenesis 2A	95716	1	274500	*TPO*	606765			1	1
		Thyroid dyshormonogenesis 1	95716	1	274400	*SLC5A5*	601843			1	1
		Pendred syndrome	705	1	274600	*SLC26A4*	605646			1	1
		Thyroid dyshormonogenesis 6	95716	1	607200	*DUOX2*	606759			1	1
		Thyroid dyshormonogenesis 5	95716	1	274900	*DUOXA2*	612772			1	1
		Thyroid dyshormonogenesis 4	95716	1	274800	*IYD*	612025			1	1
		Thyroid hormone metabolism, abnormal, 1	171706	1	609698	*SECISBP2*	607693			1	1
		Thyroid hormone metabolism, abnormal, 2	171706	1	619855	*DIO1*	147892			1	1
		Thyroid hormone metabolism, abnormal, 3	171706	1	620198	*TRU-TCA1-1*	165060			1	1
		X-linked central congenital hypothyroidism with late-onset testicular enlargement	329235	1	300888	*IGSF1*	300137			1	1
		Pituitary hormone deficiency, combined, 2	226307	1	262600	*PROP1*	601538			1	1
		Pituitary hormone deficiency, combined, 5	95494	1	182230	*HESX1*	601802			1	1
		Pituitary hormone deficiency, combined or isolated, 1	95494	1	613038	*POU1F1*	173110			1	1
		Pituitary hormone deficiency, combined, 6	226307	1	613986	*OTX2*	600037			1	1
		Deficiencia aislada de la hormona estimulante de la tiroides	90674	1	275100	*TSHB*	188540			1	1
		Hypothyroidism, congenital, nongoitrous 4	99832	1	618573	*TRHR*	188545			1	1
		Pseudopseudohypoparathyroidism	79445	1	612463	*GNAS*	139320			1	1
		Obesity, morbid, due to leptin receptor deficiency	179494	1	614963	*LEPR*	601007			1	1
		Pituitary hormone deficiency, combined, 3	95494	1	221750	*LHX3*	600577			1	1
		Pituitary hormone deficiency, combined, 4	95494	1	262700	*LHX4*	602146			1	1
		Panhypopituitarism, X-linked	90695	1	312000	*SOX3*	313430			1	1
		Hypothyroidism, congenital, nongoitrous, 8	None	1	301033	*TBL1X*	300196			1	1

↑ Increased; ↓: Decreased; #: Disease included in Basic Common Portfolio in Spain; * “Associated Disease Gene(s)” identifies the causative gene(s) for each individual disorder; ** “Recommended Gene Panel for Differential Molecular Diagnosis” lists the genes that should be considered during the molecular diagnostic evaluation initiated by an abnormal primary screening biomarker, thereby facilitating the differential diagnosis of disorders with overlapping biochemical profiles; DBS: dried blood spot; N.I.: not included; PC: primary condition; RUSP: Recommended Uniform Screening Panel from the U.S.; SC: Secondary condition; T4: Thyroxine; TSH: Thyroid-stimulating hormone.

**Table 7:** Hemoglobin disorders: Primary and secondary conditions (PC and SC) in the Spanish Uniform Screening Panel (SUSP), grouped by primary marker detected by newborn screening.

First-Tier Screening Biomarker (DBS)	Official Condition or Group of Conditions (Spanish Ministry of Health)	Condition	ORPHA Code	Consensus 1: PC 2: SC	OMIM Disease Number	Associated Disease Gene(s) *	OMIM Gene Number	Recommended Biochemical or Functional Confirmatory Tests	Recommended Gene Panel for Differential Molecular Diagnosis **	RUSP 1: PC 2: SC	Portugal 1: PC 2: SC
FS, FSC, FSa, FSD, FSE, FSOArab	Sickle cell disease #	Sickle cell disease	232	1	603903	*HBB*	141900	Hemoglobin analysis using an alternative method	*HBB*	1	1
F	B-Talasemia	Beta-thalassemia	848	1	613985	*HBB*	141900	Hemoglobin analysis using an alternative method	*HBB*	1	2
H	Hemoglobin H disease (alpha-thalassemia)	Hemoglobin H disease (alpha-thalassemia)	93616	2	613978	*HBA1*/*HBA2*	141800/141850	Hemoglobin analysis using an alternative method	*HBA1*/*HBA2*	1	2
C	Hemoglobin C disease	Hemoglobin C disease	2132	2	None	*HBB*	141900	Hemoglobin analysis using an alternative method	*HBB*	1	2
D	Hemoglobin D disease	Hemoglobin D disease	90039	2	None	*HBB*	141900	Hemoglobin analysis using an alternative method	*HBB*	1	2
E	Hemoglobin E/beta-thalassemia	Hemoglobin E/beta-thalassemia	211249	2	None	*HBB*	141900	Hemoglobin analysis using an alternative method	*HBB*	1	2

#: Disease included in Basic Common Portfolio in Spain; * “Associated Disease Gene(s)” identifies the causative gene(s) for each individual disorder; ** “Recommended Gene Panel for Differential Molecular Diagnosis” lists the genes that should be considered during the molecular diagnostic evaluation initiated by an abnormal primary screening biomarker, thereby facilitating the differential diagnosis of disorders with overlapping biochemical profiles; DBS: dried blood spot; Hemoglobin FS, FSC, FSa, FSD, FSE, FSOAra, F, H, C and D pattern; N.I.: not included; PC: primary condition; RUSP: Recommended Uniform Screening Panel from the U.S.; SC: Secondary condition.

## Data Availability

The original contributions presented in this study are included in the article. Further inquiries can be directed to the corresponding authors.

## Abbreviations

AADC	Aromatic L-amino Acid Decarboxylase (deficiency)
ACs	Autonomous Communities (Comunidades Autónomas)
AECOM	Spanish Society for Inborn Errors of Metabolism
ASMD	Acid Sphingomyelinase Deficiency
CIBERER	Spanish Network for Biomedical Research on Rare Diseases
ENS	Extended Neonatal Screening
EU	European Union
HTA	Health Technology Assessment
ISNS	International Society for Neonatal Screening
ISS	Istituto Superiore di Sanità
MLD	Metachromatic Leukodystrophy
MPS-II	Mucopolysaccharidosis type II
NBS	Newborn Screening
OMIM	Online Mendelian Inheritance in Man
ORPHAcode	Identificador de Orphanet para enfermedades raras
RUSP	Recommended Uniform Screening Panel
SUSP	Spanish Uniform Screening Panel
U.S.	United States (Estados Unidos)
WHO	World Health Organization
